# Open Education, Open Minds

**DOI:** 10.1371/journal.pbio.1000508

**Published:** 2010-10-05

**Authors:** Cheryl A. Kerfeld, Liza Gross

**Affiliations:** 1 Structural Genomics and Education Programs, United States Department of Energy, Joint Genome Institute, Walnut Creek, California, United States of America; 2 Department of Plant and Microbial Biology, University of California, Berkeley, California, United States of America; 3 Senior Science Writer/Editor, Public Library of Science, San Francisco, California, United States of America

Over the past few decades, advances in science and technology have produced a seemingly endless stream of new data, ideas, and knowledge, challenging scientists and educators alike to keep abreast of the latest concepts and discoveries in their fields. Adding to the wealth of new information posted online every day, more and more historical documents, books, and educational materials are being made available on the Web. To make the most of this treasure trove of resources, educators are increasingly sharing lessons, tools, and resources in electronic formats and online repositories [1]. Much like the open science movement, which promotes greater sharing and transparency to accelerate discovery, the open education movement freely shares fundamental and innovative teaching methods, initiatives, and materials to enhance understanding.

With this issue, *PLoS Biology* launches a new series of articles and associated resources for life sciences education. The Education Series combines the philosophy of the open education movement with the unrestricted access to scientific papers and data afforded by open-access publishing to present innovative approaches to teaching critical concepts, developments, and methods in biology. The series will cover fundamental areas of biology, including evolution and ecology, developmental biology, genomics and bioinformatics, molecular biology and genetics, immunology, microbiology, cell biology, neurobiology, and biochemistry.

Articles will showcase instructional approaches that incorporate the ideas and methods of contemporary life sciences research to help teachers engage the imagination and talents of their students. By enabling students to use the same tools researchers use and to explore real data, such approaches are especially valuable—it's widely acknowledged that engaging students in active research fosters their enthusiasm for and interest in science. Because of their accessibility through the Web, genomics databases and bioinformatics tools are especially suitable for adaptation to educational settings. Moreover, because bioinformatic algorithms can often be explained as mathematical articulations of biological concepts, articles exploring how to use such tools as BLAST provide a meaningful way to link math and science. Alternately, taking students out in the field to test relationships between the presence of contaminants in streams and the health and abundance of key species, for example, provides a memorable lesson in ecology and environmental science.

While most articles will focus on such instructional tools and approaches, we'll also feature innovative open-education initiatives and strategies that, wherever possible, draw on research reported in open-access journals. The series will take full advantage of Web-based open-access publishing and multimedia tools to create an interactive, dynamic resource for educators, researchers, and students, as well as the interested public, to enhance understanding of fundamental questions in biology and current methods to investigate them.

Although the series will focus on existing educational programs and resources, we hope authors who publish in open-access journals will consider annotating their past and future research articles with supplementary resources that can be used in the classroom or student laboratory. For example, an author might include as a supplement a student-ready laboratory or bioinformatic protocol modeled on those in the article, a set of PowerPoint slides, videotaped seminar, tutorial, or SciVee video (http://www.scivee.tv/) for teaching the context, ideas, or methods contained in the article. Collectively, as the number of articles grows, they will form a dynamic network of resources that will be available through *PLoS Biology* Collections (http://www.ploscollections.org/static/pbioCollections.action). They will be organized in various ways: according to concepts, disciplines, topics, methods, their relationship to one another, and by student level.

In 2003, The National Research Council's widely praised report on life sciences education reform, *Bio2010* [2], noted, “Outstanding textbooks such as Linus Pauling's *General Chemistry* [3] and James Watson's *Molecular Biology of the Gene* [4] have enriched and transformed undergraduate education in the past. These innovative works defined new areas of science and made them accessible and exciting to future scientists at a crucial formative stage. The need for works that sculpt science in ways that inform, enlighten, and empower the next generation of researchers is even greater today.” And, in keeping with the goals of biology education as outlined in the *Bio2010* report, we are keen to highlight those initiatives that focus on interdisciplinary connections, including those that make direct connections between the various physical and social science fields that interface with biology.

Now, in 2010, with open-access publishing, the Education Series in *PLoS Biology* can help “inform, enlighten, and empower” by providing tools and resources to every student—those in conventional courses as well as the curious child, or adult, surfing the Web for information. By providing a forum for the open exchange of educational materials, the series will provide an interactive dynamic space to share key ideas, methods, tools, and activities with our students and with the public to advance understanding of biology through the primary literature rather than textbooks. Moreover, the series provides an opportunity for the life sciences research community to get more involved in education—to have a broader impact by making your work and ideas more accessible while contributing to the development of the next generation of scientists.

By mining the promise of open education and harnessing the collective imagination and talent of *PLoS Biology* readers and contributors, the Education Series will create a virtual biology education library. In the first article, published today (doi:10.1371/journal.pbio.1000510) [5], Louise Charkoudian, Jay Fitzgerald, Andrea Champlin, and Chaitan Khosla show that Streptomyces-derived natural products provide an untapped source of useful biopigments and hope to inspire others to explore the potential of biopigments in art, industry, and perhaps most importantly, the classroom. They share their experiences in harnessing these biopigments to create paint and paintings and provide the tools for educators to replicate their experiments in the classroom. Contributions to the Education Series are encouraged; ideas should be sent to plosbiology@plos.org.
